# Etiology of bacteremias associated with c-reactive protein, procalcitonin and lactate levels

**DOI:** 10.1186/2197-425X-3-S1-A788

**Published:** 2015-10-01

**Authors:** MV de la Torre-Prados, A García-de la Torre, E Cámara-Sola, A Puerto-Morlán, T Tsvetanova-Spasova, P Nuevo-Ortega, C Rueda-Molina, A Fernández-Porcel, A García-Alcántara

**Affiliations:** Department of Intensive Care Medicine, Hospital Universitario Virgen de la Victoria, Málaga, Spain; Instituto de Investigación Biomédica de Málaga (IBIMA), Málaga, Spain; Clinical Chemistry Department, Puerto Real University Hospital, Puerto Real, Spain

## Introduction

Because of the high morbidity and mortality associated with bacteremia, prompt evaluation and adequate therapy are of paramount importance. The clinical manifestations of gram- positive and gram-negative bacterial infections are similar while biomarkers may be useful for the early diagnosis of the nature of a pathogen.

## Objectives

The purpose of the study was to evaluate the association between the level of C-Reactive Protein (CRP), procalcitonin (PCT), and lactate and the etiology of bacteremia.

## Methods

We studied the role of these biomarkers with clustered gram-positive and gram-negative bacteremia in patients hospitalized in Intensive care Unit over a period of 2 years (2011-2013), they were measured within the *24 hours* after the *onset* of severe sepsis or septic shock. The PCT was analyzed by immunoassay (Vidas, Brahms)®, lactate and CRP was measured in DIMENSION RXL - SIEMENS^®^ and blood culture was made in BACTEC-9240^®^ blood culture system (Becton Dickinson). The program used for the data processing and statistical analysis was SPSS 15.0^®.^

## Results

Our study included 396 patients, the median age of the study sample was 64 years old (inter-quartile range (IQR), 51-72), 60,6% were men, the main sources of infection were: respiratory tract (36%) and intra-abdomen (26%). In *our series, APACHE II scores* was 25 (IQR: 21-29.5), SOFA 10 (IQR: 7.75-11) and 24.8%of 28-day mortality. Blood cultures were realized in 316 patients (79.9%), 192 were negative and 7 cases the result were fungi, 58.62% had bacteremia due to gram-negative bacteria and 41.38% due to gram-positive, with 43 isolations Escherichia coli, were the most frequently isolated bacterium followed by Streptococcus pneumoniae (16.43%) and Enterobacteria (14.29%).

In the gram-negative bacteremia group, CRP levels plasma were higher 261 [IQR: 173.19-311.53] mg/dL vs. 200.2 [159.5-287.75] mg/dL than in the gram-positive bacteremia group, however PCT concentrations were statistically significant higher in the gram-negative bacteremia 27.04 [IQR: 13.47-84.23] ng/mL vs. 11.79 [2.61-22.67] ng/mL; p < 0.001; as well as lactate levels 3.1 [IQR: 1.97-4.7] mmol/L vs. 2.45 [1.64-4.22] mmol/L; p = 0.04.

## Conclusions

PCT and lactate showed differences between gram-negative and gram-positive bacteremia, might be helpful in the differentiation of pathogenic bacteriemia and supposed the etiology before obtaining blood culture results.
